# Assessing the sustainability of combined heat and power systems with renewable energy and storage systems: Economic insights under uncertainty of parameters

**DOI:** 10.1371/journal.pone.0319174

**Published:** 2025-03-18

**Authors:** Emad A. Mohamed, Mostafa H. Mostafa, Ziad M. Ali, Shady H. E. Abdel Aleem

**Affiliations:** 1 Department of Electrical Engineering, College of Engineering, Prince Sattam bin Abdulaziz University, Al Kharj 16278, Saudi Arabia; 2 Electrical Department, Faculty of Engineering, Al Ryada University for Science and Technology, Sadat City 32897, Egypt; 3 Department of Electrical Engineering, College of Engineering at Wadi Addawasir, Prince Sattam bin Abdulaziz University, Wadi Addawasir 11991, Saudi Arabi; 4 Department of Electrical Engineering, Institute of Aviation Engineering and Technology, Giza 12658, Egypt; Indian Institute of Technology Kanpur, INDIA

## Abstract

The escalating challenges posed by fossil fuel reliance, climate change, and increasing energy expenses have underscored the critical importance of optimizing energy systems. This paper addresses the economic dispatch (ED) challenge, which directs the optimization of the output of generation units to satisfy electricity and heat requirements while reducing operational expenses. In contrast to conventional economic dispatch methods, this research incorporates renewable energy sources (RESs), energy storage systems (ESSs), and combined heat and power (CHP) systems. This integrated strategy facilitates the concurrent optimization of electrical and thermal generation, culminating in a more comprehensive and efficient solution. A sophisticated scheduling model for combined heat, power, and electrical energy dispatch (CHPEED) has been devised, minimizing generation expenses. The suggested model accounts for practical constraints inherent in real-world power systems, such as prohibited operating regions, while also addressing the intricate relationships between heat and power generation in CHP units. Also, the nature of wind energy, photovoltaic systems, and load requirements within the realm of stochastic dynamic ED are considered. The general algebraic modeling system (GAMS) was utilized to solve the optimization problem. The cost without RES or ESS is $250,954.80, indicating a high reliance on costly energy sources. Integrating RES reduces costs to $247,616.42, highlighting savings through decreased fossil fuel dependency. The combination of RES and ESS achieves the lowest cost of $245,933.24, showcasing improvements in efficiency and supply-demand management via optimized energy utilization. Hence, the findings demonstrate the model’s effectiveness in addressing uncertainties associated with renewable generation, ensuring reliability in meeting energy demands and validating the possible capability to enhance the sustainability and efficiency of energy systems.

## 1. Introduction

### 1.1. Background and motivation

**Fig 1:**
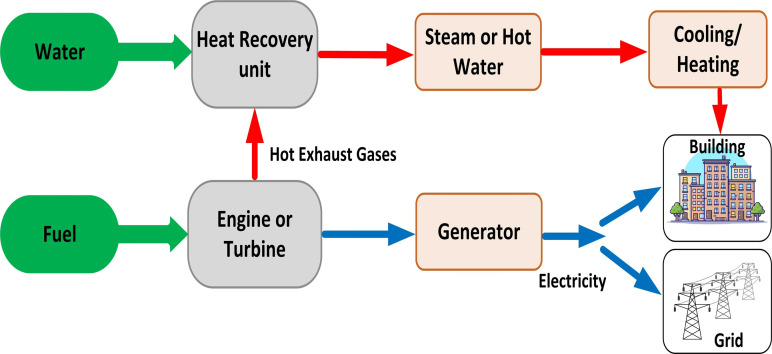
Combined heat and power system.

The increasing prominence of combined heat and power (CHP) units in power generation is attributed mainly to their ability to simultaneously deliver both electricity and heat, resulting in significant benefits for end-users and a reduction in greenhouse gas emissions [1]. In conventional thermal power plants, overall efficiency suffers considerably due to the loss of heat energy during the conversion of fuel into electricity [2]. In contrast, CHP units have the potential to significantly improve energy efficiency by effectively capturing and utilizing surplus heat [3]. Furthermore, traditional thermal power plants emit considerable amounts of harmful gases during operation, which poses serious environmental challenges [4]. In contrast, CHP units play a crucial role in reducing emissions of harmful gases, including sulfur dioxide (SO₂), nitrogen oxides (NOX), and carbon dioxide (CO₂), compared to traditional thermal power plants [5]. By integrating the utilization of heat and electricity, CHP systems significantly enhance overall power efficiency within the electricity grid. These environmentally friendly systems can achieve up to 90% energy efficiency improvements by effectively harnessing wasted heat [6]. Additionally, they have the potential to reduce harmful gas emissions by 13% to 18% while simultaneously lowering electricity generation costs by 10% to 40% [7]. Given the numerous advantages of CHP units, optimizing their operation is an essential pursuit [8]. This has led to the emergence of the economic dispatch of CHP units as a critical area of research in power system applications [9]. The combined heat, power, and electrical energy dispatch (CHPEED) optimization problem entails the coordination of three distinct types of energy production units: conventional thermal units (power-only units), CHP units, and heat-only units [10]. The primary goal of the CHPEED approach is to minimize overall system costs while fulfilling the power and heat supply requirements and adhering to pertinent system constraints [11]. These systems are designed to capture by-product heat, often steam, and utilize it effectively within the host facility, as depicted in Fig 1 [12].

The escalating levels of greenhouse gas (GHG) emissions from fossil fuel sources and their consequential impact on climate change have emerged as a pressing global concern for humanity and the environment [13]. The prevailing patterns of energy consumption worldwide, coupled with the depletion of natural resources and the energy-driven economic growth trajectory, are accelerating the pace of climate change [14]. Addressing this challenge and decarbonizing the energy system requires deploying strategic energy conversion technologies and initiatives [15]. To this end, the United Nations has delineated sustainable development goals (SDGs) with specific objectives focused on fostering a sustainable environmental and energy framework. Notably, SDG#7 underscores the imperative of integrating renewable energy systems, while SDG#13 highlights the critical need for policies to mitigate the repercussions of the climate crisis [16].

In this context, energy storage systems (ESSs) emerge as a promising technology capable of supporting system reliability, enhancing resilience to disruptions, and augmenting the integration of renewable energy sources (RESs) within electrical grids [17]. When the penetration of RESs reaches levels that jeopardize network performance, ESSs play a pivotal role in maintaining system operability [18]. Consequently, ESSs constitute essential elements within electrical networks, offering many benefits. These include improvements in power quality (PQ), heightened system reliability, increased hosting capacity for RESs, mitigation of uncertainty associated with renewable sources, enhanced system stability, reduction of power imports during peak periods by supplying peak loads and lowering the overall operational costs of the electrical network [19].

### 1.2. Literature overview

In recent literature, many works have investigated addressing combined heat and power systems with/without renewable energy resources and energy storage systems, as presented in Table 1. For instance, Paul et al. [20] introduced a pragmatic methodology utilizing chaotic driving training optimization (CDTBO) to investigate the optimal power flow (OPF) in the context of CHPEED. By incorporating chaotic-based learning with driving training optimization, the study aims to showcase the scheduling of CHPEED incorporating non-conventional energy sources while highlighting the efficacy of the CDTBO optimization technique. Gholamghasemi et al. [21] proposed using phasor particle swarm optimization for economic power generation in an economic load dispatch scenario. They considered various constraints such as transmission losses, ramp rate function, and prohibited operating zones to evaluate the effectiveness of the proposed algorithm in a real-world system setting. Jadoun et al. [22] proposed a dynamically controlled whale optimization algorithm (DCWOA) to solve multi-objective CHPEED to determine the optimal generator output of the co-generation systems, in which two conflicting objectives of the fuel cost and mass of emissions are to be simultaneously minimized. Sundaram [23] introduced a hybrid approach combining NSGA-II and MOPSO, where NSGA-II is utilized for exploration and MOPSO for the exploitation phase. A robust constraint handling strategy was implemented to effectively manage the search space within the bounds of linear and nonlinear constraints and the CHP units’ feasible operation region (FOR). Spea et al. [24] implemented an innovative optimization technique known as the social network search algorithm (SNS) to tackle the complexities of both convex and non-convex CHPEED problems. Their approach addresses real-world power system constraints, including valve loading effects, prohibited operating zones, and transmission power losses. Moreover, it considers the interdependencies between heat and power generation in CHP units. Ozkaya et al. [25] presented the dynamic switched crowding-based multi-objective symbiotic organism search (DSC-MOSOS) algorithm designed to meet the requirements and geometric constraints of the CHPEED problem. By integrating the DSC method into the MOSOS algorithm, they aimed to enhance exploration capabilities, improve the balance between exploitation and exploration, and prevent local solution entrapment. Li et al. [26] proposed a two-stage method for addressing the CHPED problem, which combines multi-objective optimization with integrated decision-making. A θ-dominance-evolutionary algorithm (θ-DEA) explored multiple Pareto optimal solutions in the first stage. The second stage involved identifying the best compromise solutions (BCSs) through the integrated decision-making (IDM) technique. Deliang et al. [27] developed a nondominated sorting genetic algorithm III with three crossover strategies (NSGA-III-TCS) to address combined heat and power dynamic economic emission dispatch (CHPDEED) problems, both with and without prohibited operating zones. Their three strategies include enhancing individuals in the first nondomination rank using a modified harmony search algorithm to facilitate quicker in-formation exchange among elite individuals, adjusting individuals in the last nondomination rank through a modified single-point crossover from genetic algorithms to improve population convergence, and utilizing a modified DE/rand-to-best/1 mutation from differential evolution to guide remaining individuals toward those with at least one minimal objective function value, thereby broadening the population’s distribution range. Hasanabadi and Sharifzadeh [28] proposed a robust methodology that utilizes a variety of mathematical transformations to tackle nonlinear and nonconvex terms in optimization problems effectively. Their approach involves converting nonconvex regions and nonlinear functions into convex polyhedrons and segments. These geometric representations are then formulated using integer variables, logical constraints, and combinatorial restrictions, resulting in a mixed-integer model that is more compatible with optimization software. Simulation results validate the effectiveness of this method, showcasing its advantages over existing solutions for CHPEED in the literature. Zou et al. [29] introduced the enhanced NSGA-II (ENSGA-II) algorithm to address CHPDEED problems. This algorithm innovatively generates offspring through a novel crossover technique based on the Cauchy distribution, incorporating adaptive location and scale parameters. This mechanism enhances the exploration and exploitation of the decision space while maintaining high population diversity. Furthermore, ENSGA-II employs a modified crowding distance metric to penalize overcrowded individuals, promoting a more uniform distribution within the objective space. A fast constraint repairing method (FCRM) is integrated to enhance its performance further, significantly reducing constraint violations and effectively guiding infeasible individuals toward feasible regions. Basu [30] introduced the squirrel search algorithm (SSA) to tackle complex multi-region CHP economic dispatch challenges, incorporating RESs. Their approach considers the valve point effect, the operational constraints of thermal generators, and the uncertainties associated with solar and wind energy. Alomoush [31] introduced an optimization technique to address the multi-objective economic-emission dispatch challenge in CHP generation within a large microgrid (μG), considering the interdependencies between heat and power outputs from CHP units and the valve-point effects of thermal units. This μG includes various fossil fuel generating units and wind and solar power units. Their approach considers operating costs, emission levels, emission taxes, and expenses related to purchasing power from the main grid. Heris et al. [32] presented an optimization technique based on the ε-constraint method for multi-objective short-term grid-connected industrial heat and power μG scheduling. This μG included a fuel cell unit, CHP units, a power-only unit, a boiler, a BSS, and a heat tank. The dispatch problem addressed cost and emissions while accounting for uncertainties and a demand response program. A fuzzy satisfying approach was employed to identify the optimal compromise solution. The study explored three scenarios: islanded mode, grid-connected mode, and the influence of the demand response (DR) program timing on μG scheduling.

**Table 1:** Overview of recent research works addressing combined heat and power systems.

Ref.	DG						Uncertainty			ESS characteristic		
CHP	Power units	Heating units	PV	WT	ESS	PV	WT	Load	Lifecycle	Lifetime	Efficiency
[20]	√	×	×	×	√	×	×	√	×	×	×	×
[21]	×	√	×	×	×	×	×	×	×	×	×	×
[22]	√	√	√	×	×	×	×	×	×	×	×	×
[23]	√	√	√	×	×	×	×	×	×	×	×	×
[24]	√	√	√	×	×	×	×	×	×	×	×	×
[25]	√	√	√	×	×	×	×	×	×	×	×	×
[26]	√	√	√	×	×	×	×	×	×	×	×	×
[27]	√	√	√	×	×	×	×	×	×	×	×	×
[28]	√	√	√	×	×	×	×	×	×	×	×	×
[29]	√	√	√	×	×	×	×	×	×	×	×	×
[30]	√	√	√	√	√	×	√	√	×	×	×	×
[31]	√	√	√	√	√	×	√	√	×	×	×	×
[32]	√	√	√	×	×	√	×	×	√	×	×	√
Proposed	√	√	√	√	√	√	√	√	√	√	√	√

Many prior studies have neglected to consider the effects of RESs and ESSs while accounting for the uncertainty of RESs and various ESS characteristics such as depth of discharge, lifetime, the number of charging/discharging cycles, and cost factors of ESS to tackle the economic dispatch challenge. This challenge involves optimizing the output of generation units to meet electricity and heat demands while minimizing operational costs. This holistic approach enables the simultaneous optimization of electrical and thermal generation, leading to a more comprehensive and efficient solution.

### 1.3. Contribution and organization

In this regard, this paper addresses the economic dispatch challenge, which directs the optimization of the output of generation units to satisfy electricity and heat requirements while reducing operational expenses. In contrast to conventional economic dispatch methods, this research incorporates RESs, ESSs, and CHP systems. This integrated strategy facilitates the concurrent optimization of electrical and thermal generation, culminating in a more comprehensive and efficient solution. A sophisticated scheduling model for combined heat, power, and electrical energy dispatch (CHPEED) has been devised, minimizing generation expenses. The general algebraic modeling system (GAMS) is used to solve the optimization problem. The model suggested accounts for practical constraints inherent in real-world power systems, such as prohibited operating regions, while addressing the intricate relationships between heat and power generation in CHP units. Also, the nature of wind energy, photovoltaic (PV) systems, and load requirements within the realm of stochastic dynamic economic dispatch are considered. In the suggested stochastic framework, parameter uncertainty is represented using the widely recognized scenario approach. This involves employing fuzzy C-means (FCM) to cluster the generated scenarios. Hence, by explicitly addressing these uncertainties, the proposed method offers a more thorough and realistic approach to power system operation and planning.

The paper is structured as follows: Section 1 presents the introduction. Section 2 describes the electrical system configuration. Section 3 details the scenario generation and reduction algorithm, i.e., uncertainty management. Section 4 presents the problem formulation and briefly overviews the GAMS model used. Section 5 presents the results obtained and their discussion. Lastly, Section 6 concludes with a summary of the research findings.

## 2. Electrical system configuration

**Fig 2:**
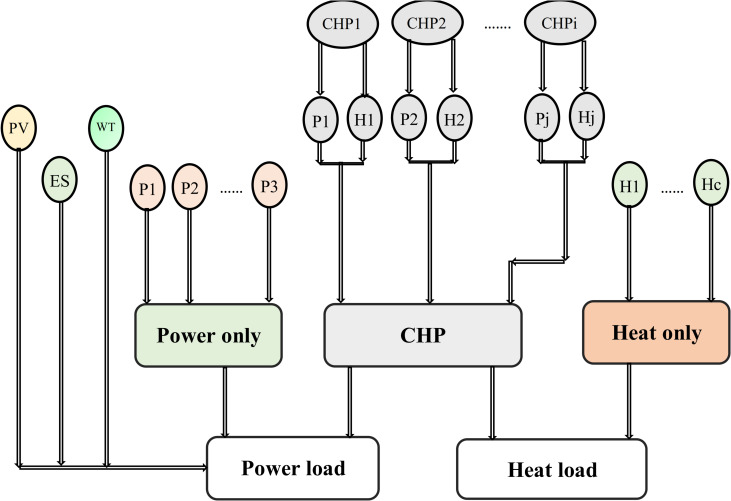
Electrical network system.

**Fig 3:**
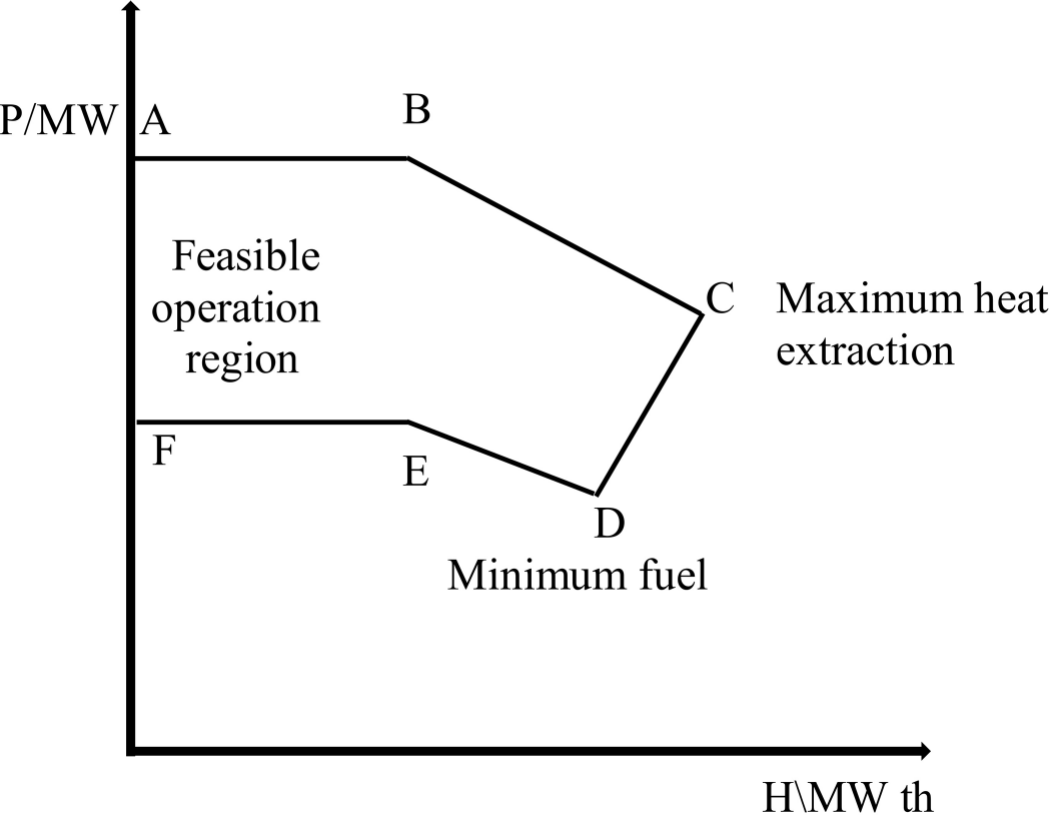
The feasible operational zone for CHP units regarding coupled heat and power generation.

RESs like solar and wind are crucial in transitioning to a sustainable energy system. However, their intermittent nature can challenge consistent energy supply [33]. To address this issue, ESSs have emerged as a vital solution, enabling the capture and storage of excess energy generated during peak production times [34]. This stored energy can then be released during periods of low generation, ensuring a stable and reliable power supply [35]. Furthermore, integrating CHP systems enhances overall efficiency by simultaneously generating electricity and proper heat from the same energy source [36]. Combining renewable energy with battery storage and CHP technologies makes it possible to create a more resilient and efficient energy system that meets electricity and heating demands while minimizing greenhouse gas emissions. Fig 2 illustrates the electrical system comprising WT, PV panels, ESS, power generation units, CHP units, and heating units utilized in applying the proposed method. The test electrical power system consists of seven units: four power generation units, two cogeneration units, and one heat unit, RESs, and ESS. The cost function parameters for each unit are detailed in Tables 2–4 [37]. Co-generation units are categorized into two types: extraction-condensing units and back-pressure units. As depicted in Fig 3, operating flexibility is limited since the back-pressure units’ electric power is directly tied to their thermal power output.

**Table 2:** Cost elements of power units.

Power units	$a_{m}$ ($/MW^2^)	$b_{m}$ ($/MW)	$c_{m}$ ($)	$\zeta_{m}$ ($)	${µ}_{m}$	Minimum limit (MW)	Maximum limit (MW)
1	0.008	2	25	100	0.042	10	75
2	0.003	1.8	60	140	0.04	20	125
3	0.0012	2.1	100	160	0.038	30	175
4	0.001	2	120	180	0.037	40	250

**Table 3:** Cost function parameters of CHP units.

CHP units	$e_{i}$ ($/MW^2^)	$g_{i}$ ($/MW)	$k_{i}$ ($)	$l_{i}$ ($/MWth^2^)	$q_{i}$ ($/MWth)	$r_{i}$ ($/MW×MWth)	FOR
5	0.0345	14.5	2650	0.03	4.2	0.031	Fig 4(a)
6	0.0435	36	1250	0.027	0.6	0.011	Fig 4(b)

**Table 4:** Cost elements of heat units.

Heat units only	$u_{s}$ ($/MWth^2^)	$v_{s}$ ($/MWth)	$z_{s}$ ($)	Minimum limit (MWth)	Maximum limit (MWth)
5	0.038	2.0109	950	0	2695.2

**Fig 4:**
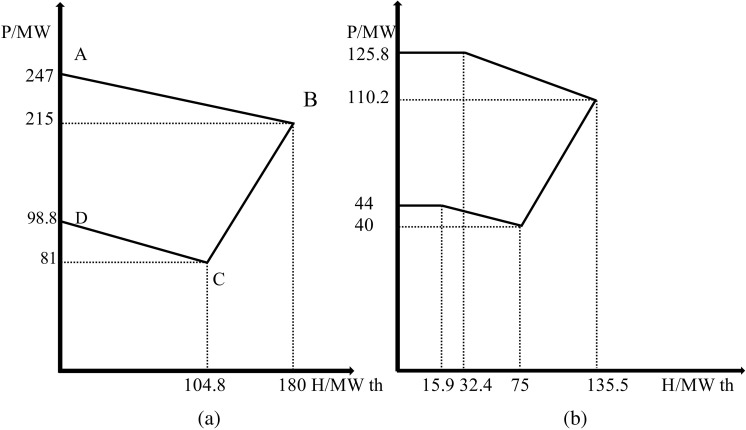
Power-heat feasible operating zone for evaluating a CHP-generating unit within the specified test power system. (a) Unit 5; and (b) Unit 6.

Fig 3 illustrates the feasible operation region of an energy system, represented on a graph with power output (P) on the vertical axis and heat extraction (H) on the horizontal axis. Points A and B indicate the boundaries of maximum fuel usage, where the system is operating at its fuel capacity. Point C marks the limit of maximum heat extraction, showing the upper boundary of heat output about power generation. The FOR area encompasses all possible combinations of power output and heat extraction that the system can achieve efficiently. Points E and F define the minimum fuel use, indicating the lower thresholds of fuel consumption while still maintaining operational effectiveness. This illustrative diagram helps understand the integrated energy system’s operational limits and capabilities [38,39]. Fig 4 shows the power-heat FOR areas for evaluating the CHP-generating units 5 and 6 within the specified test power system.

### 2.1. Wind turbine station (WTS)

A wind turbine station (WTS) harnesses the kinetic energy of wind to generate electricity. Comprising multiple wind turbines, the station converts wind movement into mechanical energy, which is then transformed into electrical energy through a generator [40]. The design of wind turbines typically includes components such as rotor blades, a nacelle housing the generator, and a tower that elevates the turbine to capture stronger winds. Wind turbine stations provide a clean energy source and help reduce greenhouse gas emissions, making them a vital part of efforts to combat climate change. Additionally, turbine technology and efficiency advancements have led to increased energy output, enhancing the overall viability of wind energy as a sustainable power source. The wind turbine station (WTSs) operates based on wind speed, which is expressed through an equation divided into four distinct parts, as shown in Eq. (1). This equation captures the relationship between wind speed and the energy output of the turbines, considering various factors that influence performance under different wind conditions [41].


$$P_{wt}^{h}= \{\begin{array}{l} 0\\ P_{wt}^{r}\left(\frac{{\left(S_{h}^{WT}\right)}^{3}-{\left(S_{cut-in}^{WT}\right)}^{3}}{{\left(S_{rated}^{WT}\right)}^{3}-{\left(S_{cut-in}^{WT}\right)}^{3}}\right)\\ P_{wt}^{r}\\ 0 \end{array}\begin{array}{cc} & \begin{array}{l} S_{WT,h}<S_{WT}^{cut-in}\;\\ S_{WT}^{cut-in}\leq S_{WT,h}<S_{RWT}\;\\ S_{RWT}\leq S_{h}^{WT}<S_{WT}^{cut-out}\\ S_{h}^{WT}\geq S_{WT}^{cut-out} \end{array} \end{array}$$ (1)

where $P_{wt}^{h}$ and $P_{wt}^{r}$ denote the output and rated powers of the WTS. $S_{WT,h}$ represents the current hour wind speed, $S_{RWT}$ is the rated wind turbine speed, $S_{WT}^{cut-out}$ represents the cut-out wind turbine speed and $S_{WT}^{cut-in}$ is the cut-in wind turbine speed.

### 2.2 Photovoltaic station (PVS)

A PV station (PVS) converts sunlight into electricity using solar panels composed of multiple photovoltaic cells. Typically located in areas with high solar insolation, these stations can significantly contribute to the renewable energy supply [42]. The energy produced can be used on-site, stored in batteries, or fed into the grid, providing flexibility in energy management. Additionally, their low environmental impact and ability to generate power without emissions align with global efforts to reduce reliance on fossil fuels and combat climate change. The output power of a PVS relies on solar radiation and atmospheric temperature, as shown in Eq. (2) [43].


$$P_{pv}^{h}=q^{PV}P_{pv}^{r}\left(\frac{O}{O_{0}}\right)\left(1-W_{c}\left(W_{a}-25\right)\right)\eta_{e}\eta_{p}$$ (2)

where $P_{pv}^{r}$ and $P_{pv}^{h}$ represent the rated power and output power of the PV system, respectively. The terms $\eta_{e}$ and $\eta_{p}$ refer to the efficiency of the inverter and the relative efficiency of the PVS. $q^{PV}$ indicates the number of PV units. $W_{c}$ and $W_{a}$ denote the temperature coefficient and the ambient temperature, respectively. Lastly, *O* and $O_{0}$ represent the global insolation and the standard solar insolation under standard test conditions.

### 2.3. Energy storage system (ESS)

ESS plays a vital role in improving the reliability and efficiency of power systems. These systems are designed to capture energy generated from renewable sources, such as solar and wind, during periods of high production and release it when demand is greater than the supply [44]. By doing so, ESS enhances grid stability, reduces reliance on fossil fuels, and allows for better integration of intermittent energy sources. Additionally, ESS can help mitigate peak load demands, providing a reliable backup during outages and contributing to overall energy efficiency. As technology advances, the capacity and affordability of ESS systems continue to improve, making them an increasingly vital component of sustainable energy solutions [45]. They can be categorized into several types based on their technology and application. Battery energy storage is the most prevalent form, utilizing various technologies such as lithium-ion, lead-acid, and flow batteries. These systems are well-suited for short- to medium-term storage, offering quick response times and high efficiency. Pumped hydro storage (PHS) is another traditional method, where water is pumped to a higher elevation during low demand and released to generate electricity during peak times, providing large-scale storage with long discharge durations. Flywheel energy storage systems store kinetic energy and can deliver high power output for short periods, making them ideal for applications that require rapid response and frequency regulation. Thermal energy storage involves storing energy as heat, often using materials like water or molten salt, and is commonly utilized in concentrated solar power plants. Lastly, compressed air energy storage (CAES) systems store energy by compressing air in underground caverns, releasing it to drive turbines during peak demand. Each type of ESS has distinct advantages and is suitable for varying requirements in the energy grid, including capacity, duration, and response time [46,47]. The capital cost of the ESS (denoted as $CC_{ES}$) is tied to its power capacity ($P^{ES}$) and energy capacity ($E^{ES}$), as expressed in Eq. (3) [48].


$$CC_{ES}=\left(CP^{ES}\times P^{ES}\right)+\left(CE^{ES}\times E^{ES}\right)$$ (3)

where $CP^{ES}$and $CE^{ES}$represent the cost coefficients for the ESS based on its rated power and energy capacities.

## 3. Uncertainty management

The uncertainty associated with WT and PV systems and electrical loads poses significant challenges in energy management. Both wind and solar energy generation are inherently variable due to fluctuating weather conditions, which can lead to unpredictable power output. For instance, wind speed can change rapidly, affecting turbine performance, while solar energy production can be influenced by cloud cover and seasonal variations. Similarly, electrical loads can vary based on time of day, weather, and consumer behavior, making it difficult to forecast demand accurately [49]. This unpredictability necessitates advanced forecasting methods and flexible grid management strategies to ensure a stable energy supply. Addressing these uncertainties is crucial for optimizing the integration of renewable energy sources and maintaining grid reliability [50]. In this research, several scenarios are developed to illustrate the uncertainties in different system parameters, each accompanied by a defined probability. FCM generates uncertain electricity demand, RES production, and market pricing scenarios. Additionally, FCM assists in clustering these scenarios into a more manageable subset.

In this study, as detailed in [51] and [52], FCM is employed to partition a dataset of size *S* into a fixed number of clusters *C*, specifically set to 5. As the number of scenarios increases, the complexity of the task escalates, requiring more extensive computational resources. The data for clustering is organized in a matrix *Q*, composed of column vectors $Q_{j}$ for j=1,2,…,S. FCM requires two parameters for clustering: the number of clusters *C* and the fuzziness parameter *e*, where r>x and r>1. A predefined tolerance level *ϵ* is established to determine when to terminate the clustering process. The FCM algorithm follows five distinct steps:

1. Initialization: A membership matrix $x={\left[x_{ij}\right]}_{C\times S}$ is randomly initialized, ensuring that the sum of each column *j* equals 1. *C* random centroids are selected from the dataset, forming a vector =  ${\left[C_{i}\right]}_{1\times C}$;
2. Centroid calculation: New centroids are computed using the formula:

$$C_{i}=\frac{\sum_{i=1}^{S}x_{ij}^{r}\times Q_{j}}{\sum_{i=1}^{S}x_{ij}^{r}}$$ (4)

3. Membership matrix update: The elements of the membership matrix $\left(x={\left[x_{ij}\right]}_{C\times S}\right)$ are recalculated for each element in *Q* using:

$$x_{ij}=\frac{1}{\sum_{p=1}^{C}{\left(\frac{Q_{j}-C_{i}}{Q_{j}-C_{p}}\right)}^{\frac{2}{r-1}}}$$ (5)

4. Objective function evaluation: The objective function value at the $it^{th}$iteration is computed as:

$$f_{FCM}^{\left(it\right)}=\overset{S}{\underset{j=1}{\sum }}\overset{C}{\underset{i=1}{\sum }}x_{ij}^{r}Q_{j}-C_{i}$$ (6)

5. Convergence check: If the following difference is less than *ϵ*; for all it, the optimization process prints the results; otherwise, the procedure is repeated from Stage 2.

$$f_{FCM}^{\left(it\right)}-f_{FCM}^{\left(it-1\right)}<\epsilon ,\forall it$$ (7)

**Fig 5:**
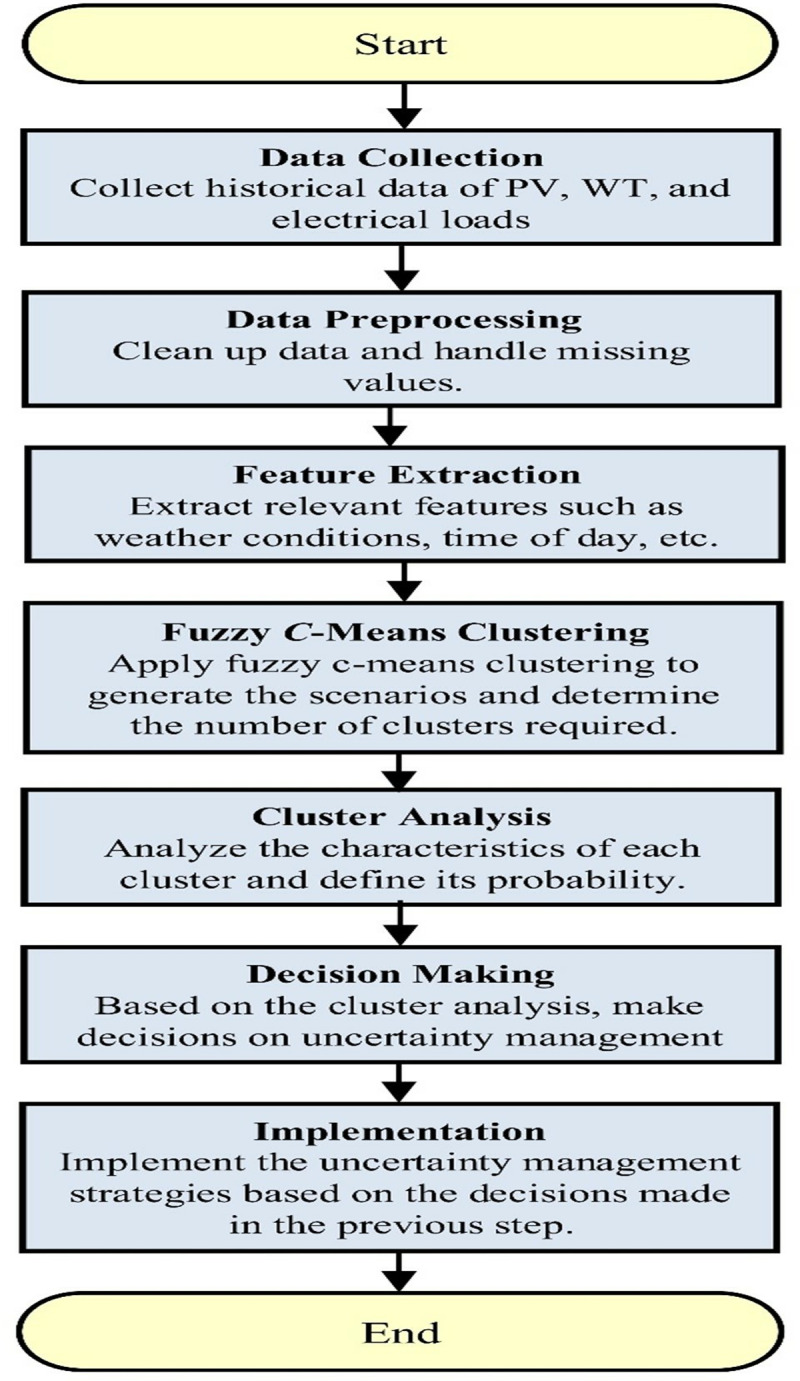
Flowchart for uncertainty management of PV, WT, and electrical load.

Fig 5 shows a flow chart for uncertainty management of PV, WT, and electrical load. The flowchart details the process for managing uncertainty in a system involving PV, WT, and electrical load data. It begins with collecting historical data on PV, WT, and electrical loads, followed by data preprocessing to clean and address missing values. Subsequently, relevant features like weather conditions and time of day are extracted in the feature extraction stage. The process then applies fuzzy *C*-means clustering to generate scenarios and determine the necessary number of clusters. Cluster analysis is then conducted to understand the characteristics of each cluster and establish their probabilities.

Based on this analysis, decisions regarding uncertainty management strategies are made. Finally, the chosen strategies are implemented in the system as per the decisions made in the previous step. This flowchart outlines a structured approach to handling uncertainty in a system reliant on PV, WT, and electrical load data, ensuring a methodical and informed decision-making process.

## 4. Problem formulation and optimization platform

**Fig 6:**
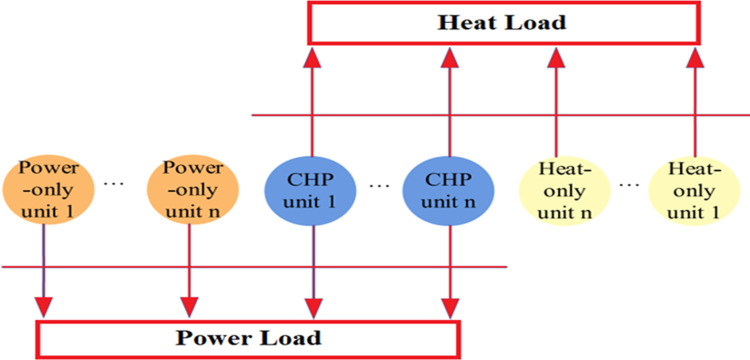
The basic structure of the CHP system.

A CHP system comprises power-only, CHP, and heat-only units, as depicted in Fig 6. This study aims to identify the best output configurations by considering power and heat demand constraints.

### 4.1. Objective function

The primary goal of CHPEED’s objective function is to reduce overall operational expenses. These expenses encompass the costs associated with heat-only $\left(f_{w}\left(H_{w,h}^{H}\right)\right)$ and power-only units $\left(f_{m}\left(P_{m,h}^{p}\right)\right)$, CHP systems $\left(f_{n}\left(P_{n,h}^{c},H_{n,h}^{c}\right)\right)$, generation expenses for WT ($P_{wt}^{h}$) and PV ($P_{pv}^{h}$) systems, as well as the daily total expenses for ESS $\left(TCES\right)$, considering multiple scenarios of PV output power (PVS) along with their respective probabilities ($\alpha_{s,pv}^{h}$), and various scenarios of WT output power (WTS) along with their probabilities ($\beta_{s,wt}^{h}$.). The anticipated operational costs $\left(f\left(x\right)\right)$ can be expressed as:


$$f\left(x\right)=\overset{H}{\underset{h=1}{\sum }}[\overset{MP}{\underset{m=1}{\sum }}\left(f_{m}\left(P_{m,h}^{p}\right)\right)+\overset{NC}{\underset{n=1}{\sum }}\left(f_{n}\left(P_{n,h}^{c},H_{n,h}^{c}\right)\right)+\overset{WH}{\underset{w=1}{\sum }}\left(f_{w}\left(H_{w,h}^{H}\right)\right)+\overset{PVS}{\underset{s=1}{\sum }}\left(\alpha_{s,pv}^{h}\times C_{pv}^{h}\times P_{pv}^{h}\right)+\overset{WTS}{\underset{s=1}{\sum }}\left(\beta_{s,wt}^{h}\times C_{wt}^{h}\times P_{wt}^{h}\right)+TCES$$ (8)

where NC, MP, and WH represents the number of units of CHP units, the power-only units, and the heat-only units, respectively. $C_{pv}^{h}$, and $C_{wt}^{h}$ are the kWh cost of WT and PV at each hour *h*, respectively. Also [37],


$$f_{m}\left(P_{m,h}^{p}\right)=a_{m}{\left(P_{m,h}^{p}\right)}^{2}+b_{m}\left(P_{m,h}^{p}\right)+c_{m}$$ (9)

$$f_{n}\left(P_{n,h}^{c},H_{n,h}^{c}\right)=e_{n}{\left(P_{n,h}^{c}\right)}^{2}+g_{n}\left(P_{n,h}^{c}\right)+k_{n}+l_{n}{\left(H_{n,h}^{c}\right)}^{2}+q_{n}\left(H_{n,h}^{c}\right)+r_{n}P_{n,h}^{c}H_{n,h}^{c}$$ (10)

$$f_{s}\left(H_{s,h}^{H}\right)=u_{s}{\left(H_{s,h}^{H}\right)}^{2}+v_{s}\left(H_{s,h}^{H}\right)+z_{s}$$ (11)

where $a_{m},b_{m},c_{m}$ are the cost coefficients related to the power-only units. $e_{n},g_{n},k_{n},l_{n},q_{n},r_{n}$ are the cost coefficients related to CHP units. $u_{s},v_{s},z_{s}$ are the cost coefficients related to heat-only units.

The expenses associated with ESS encompass various components, including the initial capital investment and the costs related to replacements throughout the project’s lifespan. The capital cost of the BSS (denoted as $CC_{ES}$) is tied to its power capacity ($P^{ES}$) and energy capacity ($E^{ES}$), as expressed in Eq. (3). To calculate the number of replacements needed for the ESS, we first determine the number of cycles completed ($ES_{cy}$) using Eq. (12) and (13) to evaluate the ESS’s lifespan. Thus, Eq. (14) is employed to find the lifespan of the ESS ($S_{ES}$), which is based on the lifecycle of the ESS ($ES_{Lifecycles}$) and $ES_{cy}$ [40].


$$N_{ES}\left(h,i\right)=\left(Q_{w\left(t\right)}-Q_{w\left(h-1\right)}\right)R_{w\left(h\right)},\forall h\in H,\forall i\in T$$ (12)

$$ES_{cy}=\overset{T}{\underset{i=1}{\sum }}\overset{H}{\underset{h=1}{\sum }}N_{ES}\left(h,i\right)$$ (13)

$$S_{ES}=\frac{ES_{Lifecycles}}{ES_{cy}}$$ (14)

where $N_{ES}\left(h,i\right)$ denotes the ESS cycles, *T* indicates the total operating days each year and $Q_{w\left(t\right)}$ reflects the operational status of the ESS for each hour during those days.

Consequently, the total number of replacements ($R_{ES}$) for the ESS over the project’s duration (ℜ) is given by Eq. (15).


$$R_{ES}=\frac{ℜ}{S_{ES}}$$ (15)

Finally, the total cost of the ESS system (TCS, in $/day) can be calculated using Eq. (16), where i is the interest rate:


$$TCS=\frac{1}{T\times }\left(\frac{i{\left(1+i\right)}^{}}{{\left(1+i\right)}^{}-1}\times CC_{ES}\times R_{ES}\right)$$ (16)

### 4.2. Constraints

The study must incorporate several constraints to ensure the solution is feasible.

#### 4.2.1. Power-only units’ constraint.

The power output of each power-only unit must work within its defined capacity at any given time, ensuring that the operational limits of each unit are respected across the entire time horizon, as detailed in Eq. (17) [37].


$$P_{m,h}^{p,min}\leq P_{m,h}^{p}\leq P_{m,h}^{p,max}\forall m�\text{MP},\forall h$$ (17)

#### 4.2.2. Heat-only units’ constraint.

The heat output of each heat-only unit must work within its defined capacity at any given time, ensuring that the operational limits of each unit are respected across the entire time horizon, as detailed in Eq. (18) [37].


$$H_{s,h}^{H,min}\leq H_{s,h}^{H}\leq H_{s,h}^{H,max},\forall h$$ (18)

#### 4.2.3. CHP units’ constraint.

CHP units are subject to several constraints that are essential for their efficient and reliable operation. First, the power output must remain within defined minimum and maximum levels to ensure the unit operates within its design capabilities. Additionally, the thermal output generated by the CHP must align with the heat demand of the connected load, ensuring that enough heat is produced without excess generation, considering the limits of CHP units’ thermal output [37].


$$P_{n,h}^{C,min}\left(H_{n,h}^{C}\right)\leq P_{n,h}^{C}\leq P_{n,h}^{C,max}\left(H_{n,h}^{c}\right),\forall n�\text{NC},\forall h$$ (19)

$$H_{n,h}^{C,min}\left(P_{n,h}^{C}\right)\leq H_{n,h}^{C}\leq H_{n,h}^{C,max}\left(P_{n,h}^{C}\right),\forall n�\text{NC},\forall h$$ (20)

#### 4.2.4. WT and PV constraints.

The output power generated by the WT is constrained by its minimum power level, denoted as $P_{wt,min}^{h}$, and its maximum power level, represented as $P_{wt,max}^{h}$, as depicted in Eq. (21). Likewise, the power output from the PVS is limited by its minimum power value, $P_{pv,min}^{h}$, and its maximum power value, $P_{pv,max}^{h}$, as depicted in Eq. (22) [48].


$$P_{wt,min}^{h}\leq P_{wt}^{h}\leq P_{wt,max}^{h},\forall h$$ (21)

$$P_{pv,min}^{h}\leq P_{pv}^{h}\leq P_{pv,max}^{h},\forall h$$ (22)

#### 4.2.5. Power generation-demand equilibrium.

The total output power generated by the power-only units, CHP units, RESs, and ESS must match the total electrical demand scenarios ($P_{ED,s,h}$) and their associated probabilities (${\text{µ}}^{s,h}$) throughout the day, as depicted in Eq. (23).


$$\overset{MP}{\underset{m=1}{\sum }}P_{m,h}^{p}+\overset{NC}{\underset{n=1}{\sum }}P_{m,h}^{p}+\overset{PVS}{\underset{s=1}{\sum }}\left(\alpha_{s,pv}^{h}\times P_{pv}^{h}\right)+\overset{WTS}{\underset{s=1}{\sum }}\left(\beta_{s,wt}^{h}\times P_{wt}^{h}\right)+P_{discharge,h}^{ES}=\overset{ED}{\underset{s=1}{\sum }}{µ}^{s,h}\times P_{ED,s,h})+P_{charge-h}^{ES},\forall h\in T$$ (23)

where $P_{discharge,h}^{ES}$ and $P_{charge-h}^{ES}$ represent the discharge and charge of the ESS, respectively.

#### 4.2.6. Energy storage limits and bounds.

ESS has several limitations that must be taken into account in this study, including $P_{charge-h}^{ES}$ and $P_{discharge,h}^{ES}$, as outlined in Eq. (24) and (25) [40]:


$$P_{charge-h}^{ES}\leq P_{charge-h}^{ES-max},\forall h\leq T$$ (24)

$$P_{discharge-h}^{ES}\leq P_{discharge-h}^{ES-max},\forall h\leq T$$ (25)

The state of charge (SoC) of the ESS, denoted as $SoC^{ES,h}$, must be within the specified minimum ($SoC_{min}^{ES,h}$) and maximum ($SoC_{max}^{ES,h}$) limits, as depicted in Eq. (26). The current $SoC^{ES,h}$ is a function of the previous $SoC^{h-1}$ along with the charge and discharge capacities at the current time h as described in Eq. (27). The initial SoC of ESS $\left(SoC^{in}\right)$ is established at the starting time (*h = 1*) as described in Eq. (28). At the end of the day, $SoC^{ES,h}$ must equal the initial SoC to ensure that it remains constant [40].


$$SoC_{min}^{ES,h}\leq SoC^{ES,h}\leq SoC_{max}^{ES,h},\forall h\leq H$$ (26)

$$SoC^{ES,h}= \{\begin{array}{c} SoC^{in}+�h\eta^{ES}P_{charge-h}^{ES}-�hP_{discharge-h}^{ES},h=1\\ SoC^{h-1}+�h\eta^{ES}P_{charge-h}^{ES}-�hP_{discharge-h}^{ES},\forall h\geq 2,h\in T \end{array}$$ (27)

$$SoC^{ES,h}=SoC^{in},h=24$$ (28)

Eq. (29) indicates that, at any given time, the total discharge power equals the total charge power, adjusted for the efficiency of ESS ($\eta^{ES}$); thus [40]:


$$\overset{T}{\underset{h=1}{\sum }}P_{discharge,h}^{ES}=\overset{T}{\underset{h=1}{\sum }}P_{charge-h}^{ES}\times \eta^{ES}$$ (29)

## 4. General algebraic modeling system (GAMS)

GAMS is a high-level modeling system designed for mathematical programming and optimization problems. It provides a powerful framework for formulating complex models. GAMS allows users to express their models clearly and concisely using algebraic notation, which can then be solved using a variety of optimization solvers [53]. Its flexibility and scalability make it suitable for tackling large-scale problems involving linear, nonlinear, and mixed-integer programming. GAMS supports various optimization solvers, enhancing its versatility for various modeling applications [54]. Notable among these is CPLEX, a robust solver adept at handling linear, mixed-integer, and quadratic programming problems. Gurobi is another high-performance option, particularly favored for its efficiency in solving large-scale linear and mixed-integer models. MINOS and CONOPT are commonly used for nonlinear optimization, with the former focusing on smooth nonlinear problems and the latter on large-scale nonlinear optimization. BARON excels in mixed-integer nonlinear programming, while KNITRO is recognized for its capabilities in both constrained and unconstrained nonlinear scenarios. Additionally, SCIP is an open-source solver ideal for mixed-integer programming, and LINDO offers a range of solutions for linear and nonlinear challenges. The COIN-OR project provides a collection of open-source solvers, further expanding the options available to GAMS users. This compatibility with multiple solvers allows operators to select the most appropriate tools for their specific optimization needs [55]. Some researchers prefer GAMS over MATLAB for optimization tasks due to its high-level modeling language, which allows for clearer and more abstract formulations of complex problems. It provides flexibility with a wide range of specialized solvers suited for linear, nonlinear, and mixed-integer problems, making it ideal for large-scale optimization often encountered in operations research. GAMS supports modular programming, enhancing model management and updates, and includes robust features for sensitivity analysis and scenario planning. Its efficiency in handling large datasets and sparse matrices, coupled with extensive documentation and a strong user community, further solidifies its appeal. While MATLAB is a powerful general computing environment, GAMS’s specialization in optimization makes it a more efficient choice for users. Within the GAMS environment, various nonlinear programming (NLP) solvers are available, including MINOS, IPOPT, SNOPT, and KNITRO. However, the choice to use the embedded CONOPT solver in this study was influenced by several key factors: CONOPT is particularly effective for models with highly nonlinear constraints, can handle problems that involve second derivatives, and incorporates logic that dynamically alternates between sequential linear programming and sequential quadratic programming relying on the current needs of the solution process. Additionally, CONOPT is specifically designed for large-scale nonlinear models featuring smooth functions, although it can accommodate those with non-differentiable elements [56].

**Fig 7:**
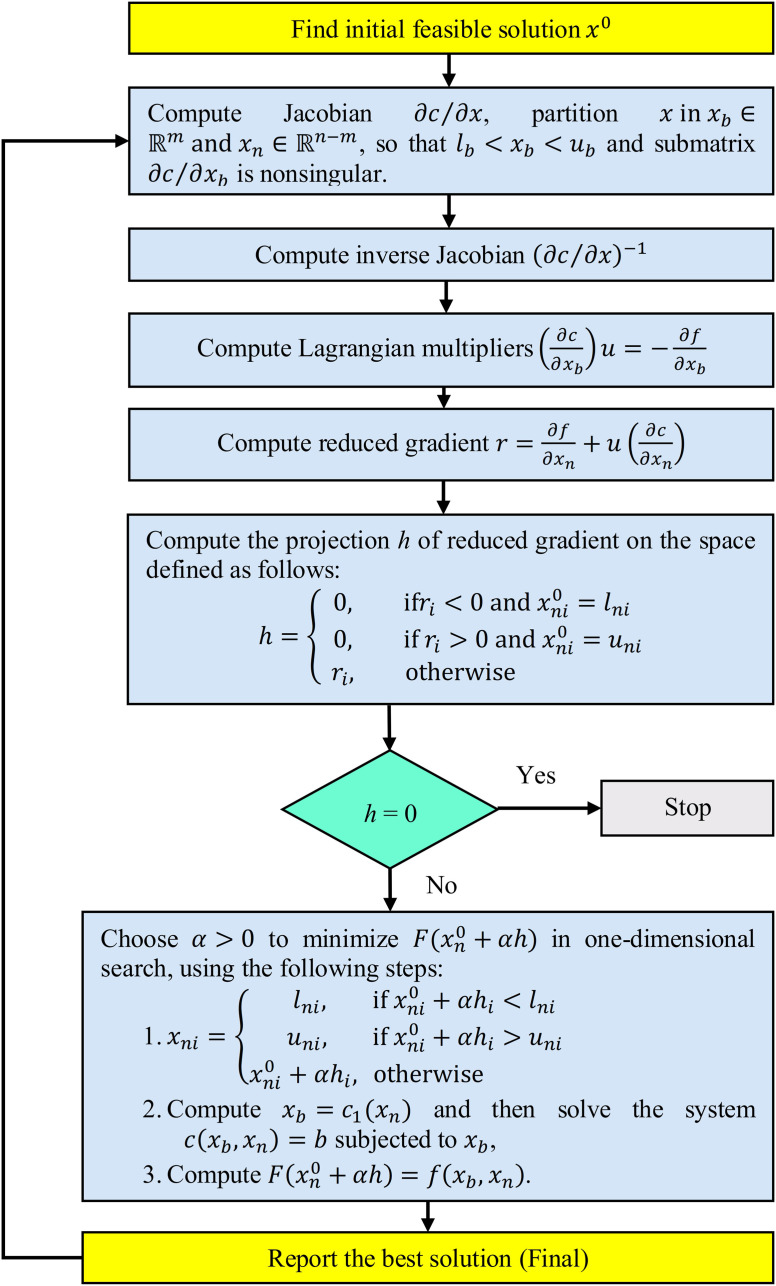
CONOPT solver algorithm on the GAMS platform.


To tackle complex nonlinear optimization problems with sparse and nonlinear constraints, the generalized reduced gradient method, particularly the CONOPT approach, is often utilized. CONOPT combines techniques from sequential linear programming and sequential quadratic programming with generalized reduced gradients to find optimal solutions for both static and dynamic models. Its flowchart is depicted in Fig 7. It proceeds by addressing the general nonlinear optimization problem, formally defined as follows [57,58]:

$$\min f\left(x\right)$$ (30)

subjected to:


$$c\left(x\right)=b,\;l\leq x\leq u$$ (31)

In this formulation, $x\in {\mathbb{R}}^{n}$ represents a vector of *n* variables $x_{i}$; $f:{\mathbb{R}}^{n}\to \mathbb{R}$ represents a real-valued objective function; $c:{\mathbb{R}}^{n}\to {\mathbb{R}}^{m}$ defines equality constraints; $b\in {\mathbb{R}}^{m}$ establishes a vector of *m* bounds for these constraints and $l,u\in {\mathbb{R}}^{n}$ are vectors of *n* simple bounds for the variables *x*.

## 5. Results and discussions

### 5.1. System data

**Fig 8:**
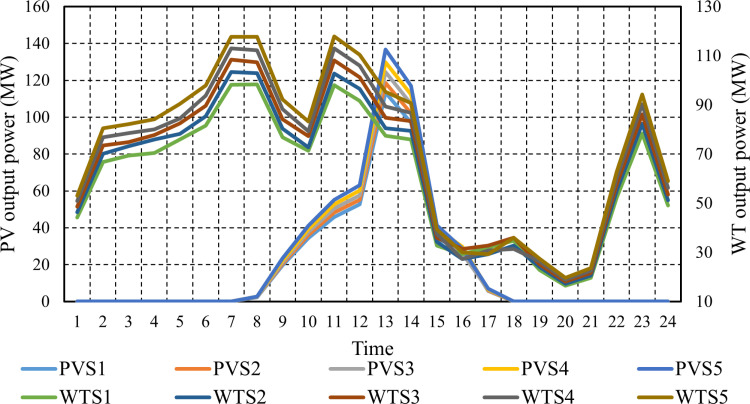
Hourly power scenarios of PVS and WST.

The stochastic method addresses uncertainty parameters by utilizing multiple scenarios with associated probabilities. This approach aims to enhance the comprehension of how parameter uncertainty influences outcomes. To achieve this, 1000 scenarios are generated based on historical data to represent the uncertainties related to PV systems, WT, and load demand. Next, a scenario reduction method is implemented, employing the FCM clustering algorithm to consolidate the PVS, WTS, and demand scenarios into the five most likely scenarios. This reduction process is crucial for optimizing computational efficiency. Following this reduction, Fig 8 illustrates the clustered power output of PV and WT stations.

**Fig 9:**
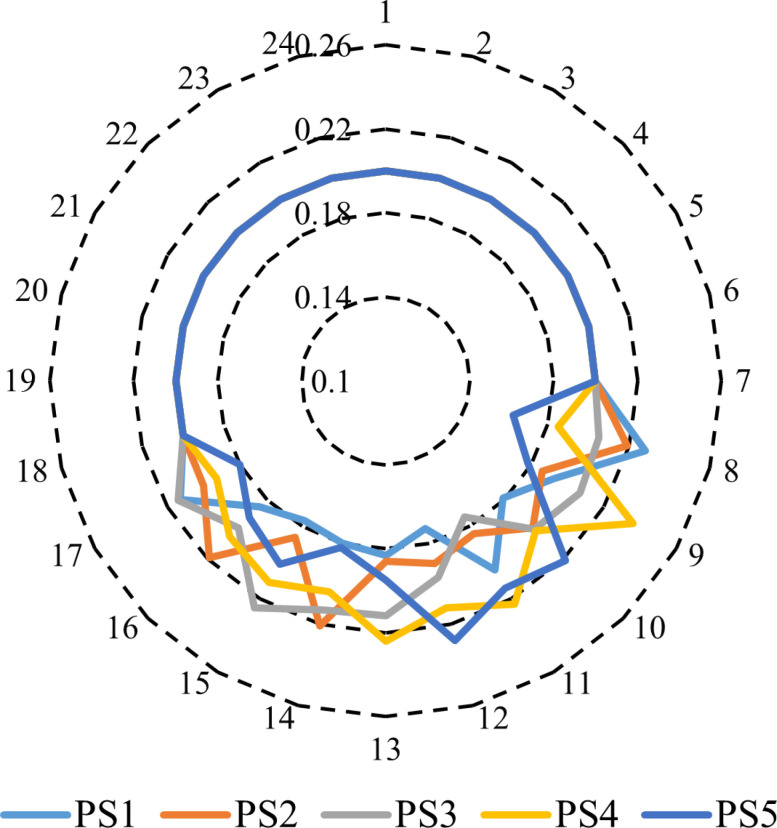
Probability of power scenarios of PVS.

**Fig 10:**
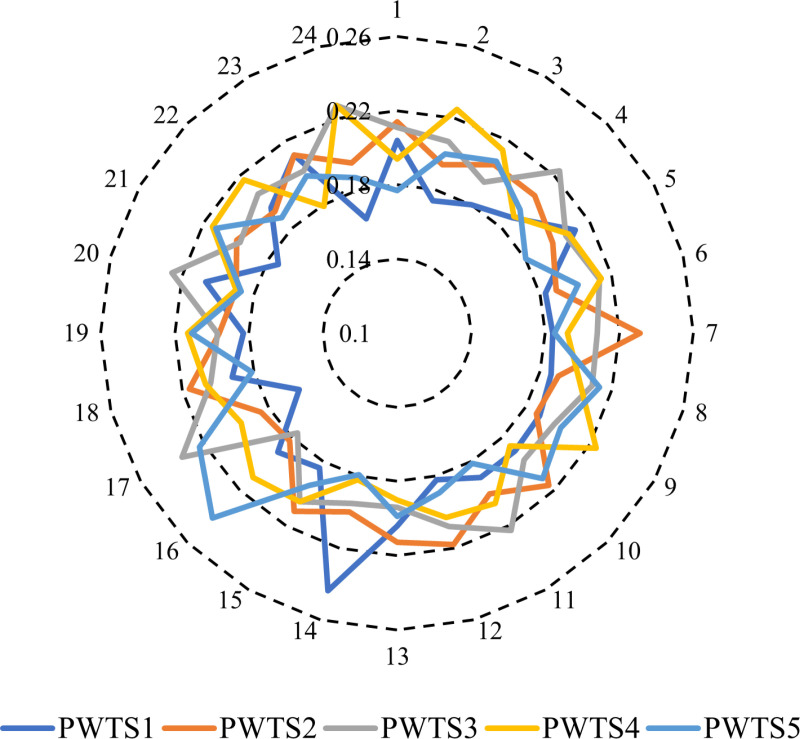
Probability of power scenarios of WTS.

The respective probabilities of the clustered power output of PV are shown in Fig 9. The respective probabilities of the clustered power output of WTS are demonstrated in Fig 10. The cost of electricity produced by a WTS is $.08 per kWh, while the cost of electricity generated by PVS is $ 0.12 per kWh [59].

**Fig 11:**
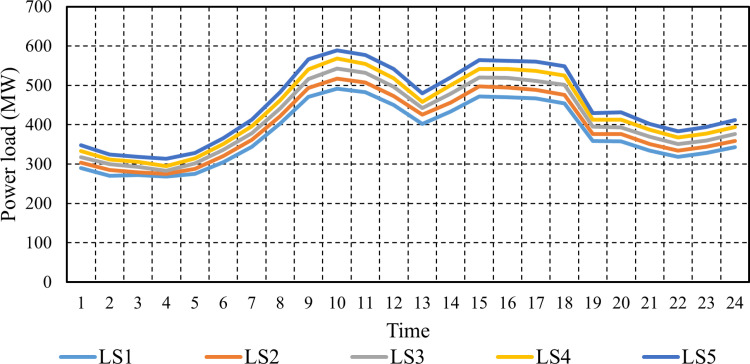
Hourly generated electrical load scenarios.

**Fig 12:**
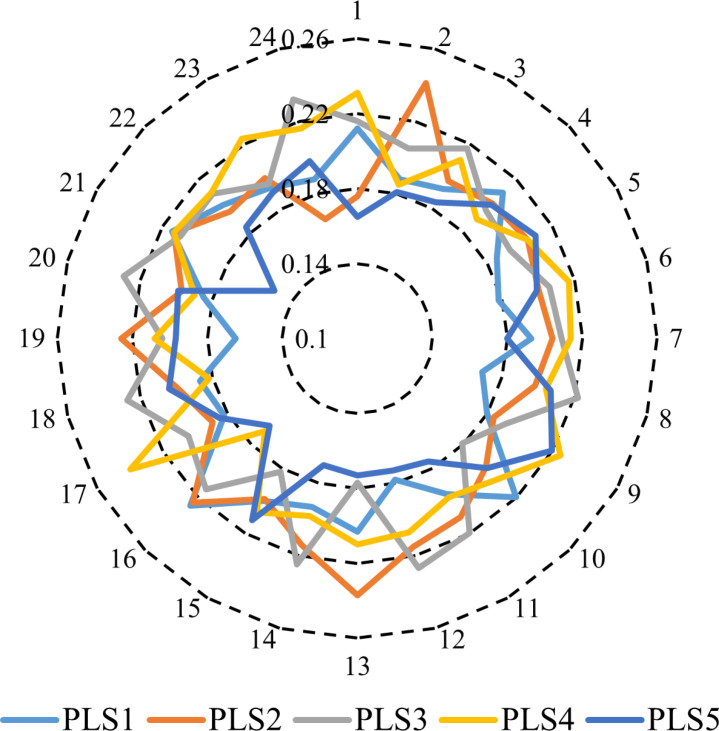
Probability of power scenarios of power demand.

The power demand in the electrical grid studied is 600 MW, and the heat demand is 300 MWth [37]. It is essential to note that the electricity load is dynamic, varying from hour to hour. In the investigated electrical network, the maximum load stands at 600 MW, with the load at each hour being represented as a proportion of this peak value. The inherent variability in user demand contributes significantly to the complexity of electrical system management, emphasizing the importance of considering electrical load uncertainties in this study. The scenarios of electricity load clustering for each hour of the day are depicted in Fig 11, with the corresponding probabilities depicted in Fig 12.

The electrical grid under investigation exhibits a power demand of 600 MW and a heat demand of 300 MWth [37]. Notably, the electricity load is dynamic, exhibiting variations on an hourly basis. The inherent fluctuations in user demand introduce considerable complexity to the management of the electrical system, underscoring the necessity of accounting for uncertainties associated with the electrical load in this study. Addressing these uncertainties using a stochastic method involves employing multiple scenarios with associated probabilities to elucidate the impact of parameter uncertainty on outcomes. To this end, 1000 scenarios are derived from historical data to represent the uncertainties inherent in load demand. Subsequently, a scenario reduction technique is applied, utilizing the FCM clustering algorithm to condense the demand scenarios into the five most probable scenarios. This reduction process plays a pivotal role in enhancing computational efficiency.

Following this reduction, Fig 11 visually represents the clustering of electricity load for each hour of the day, with the corresponding probabilities depicted in Fig 12. This methodology enables a comprehensive exploration of parameter uncertainties and their implications on system performance, facilitating informed decision-making processes within the electrical grid.

The following subsections present and discuss the results obtained for the optimal CHPEED of the analyzed electrical system in three scenarios. The base case (Case 0), the optimal CHPEED is conducted without considering RESs and ESS. In the first case (Case 1), the optimal CHPEED is carried out with the inclusion of RESs. In the second case (Case 2), the optimal CHPEED is performed with RESs and ESS.

In addition, GAMS provides a flexible and powerful platform for modeling complex decision-making problems. In GAMS, users can define optimization models using algebraic equations, variables, and constraints. These models can encompass linear, nonlinear, integer, and mixed-integer programming formulations. GAMS facilitates the integration of these models with various solvers to find optimal solutions efficiently [55]. When evaluating the performance of an optimization model in GAMS, several key criteria are typically considered [55]:

1) Solver status (Return code)

The solver status indicates whether the optimization problem was solved successfully or if errors occurred during the solution process. A return code of 1 signifies that the problem was solved without errors.

2) Model status

The model status provides information about the quality of the solution obtained by the solver. A model status of 1 indicates that the solution found is a global optimum, which is the best possible solution given the problem formulation and constraints.

In this study in all cases, the solver status returns 1 in the solution report of GAMS/ CONOPT, which means that the problem is solved without errors. Also, the model status is 1 which means that the solution to the problem is a global solution.

In GAMS, checking for constraint violations involves defining your model with variables and equations, and then solving it using the SOLVE statement. After solving, you can assess the feasibility of constraints by comparing the left-hand side (LHS) and right-hand side (RHS) of each constraint. This is done by calculating the difference for each constraint and displaying these values. If any calculated violation is greater than zero, it indicates a constraint violation, while a value of zero or negative means the constraints are satisfied. This process allows for effective monitoring and adjustment of your model to ensure all specified constraints are adhered to. GAMS generates a log file during execution that contains detailed information about the model run, including warnings or errors related to constraint violations [55]. The report on GAMS constraint violations in our study indicates that there were no violations of the constraints taken into account.

### 5.2. Case 0

**Fig 13:**
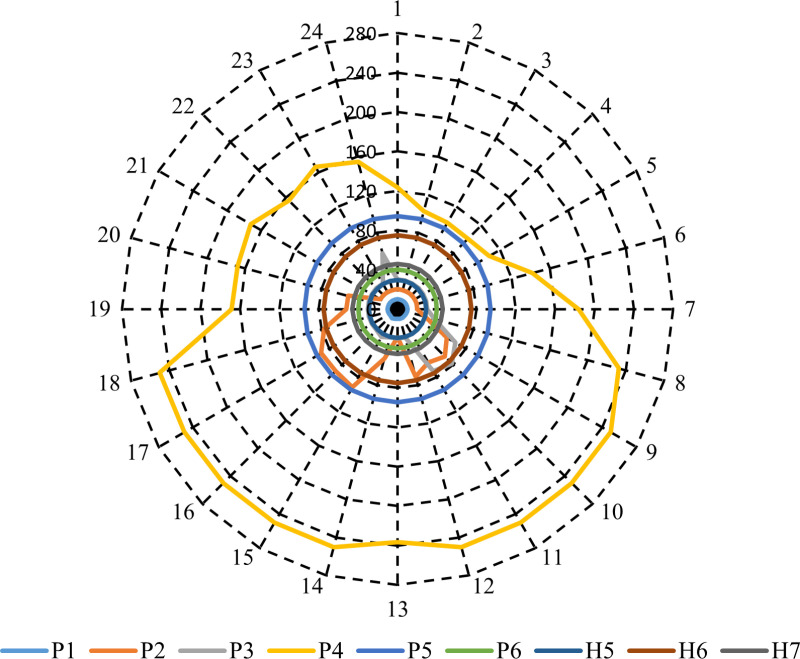
The economic dispatch for power-only units, CHP systems, and heat-only units in Case 0.

In this case, the optimal CHPEED is carried out independently of RESs and ESS. Fig 13 shows the optimal output of CHP units at each hour throughout the day to achieve minimum operation cost. It can be seen from Fig 13 that the output power of the sources P1 to P6 varies at each hour depending on the electrical demand and the operational constraints of each unit, with some sources maintaining steady output throughout the day while others exhibit fluctuations. For instance, P1 shows consistent power output at its minimum output power (10 MW) throughout all hours. P2 and P3 show varying power outputs, with peaks aligning with hours of heavy power loads. P4, on the other hand, demonstrates significant fluctuations in power generation, reaching a maximum output of 250 units in several hours. Notably, the heat demands were divided between the CHP units (H5, H6) and the heat-only unit (H7) throughout the 24 hours. It can be seen from Fig 13 that H5, H6, and H7 remain constant at 29.2 MWth, 75MWth, and 45.7MWth, respectively, throughout the 24 hours. In this case, the obtained results show that the overall operation cost of the studied electrical system is $250954.7.

### 5.3. Case 1

**Fig 14:**
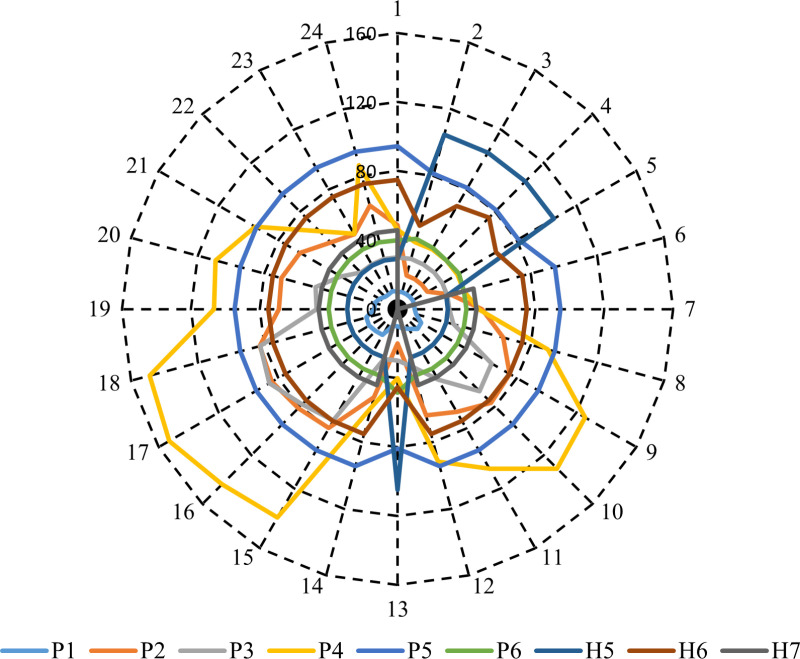
The economic dispatch for power-only units, CHP systems, and heat-only units in Case 1.

In this case, the optimal CHPEED is carried out with the inclusion of RESs. Fig 14 shows the optimal output of CHP units at each hour throughout the day to achieve minimum operation cost after including PV units and the WT system. It can be seen from Fig 14 that the output power of the sources P1 to P6 varies at each hour depending on the electrical demand, output power of PV and WT, and the operational constraints of each unit, with some sources maintaining steady outputs throughout the day while others exhibit fluctuations.

For instance, P1 maintains a consistent value of around 10 MW for most hours, slightly increasing during hours 8 to 10 due to heavier load conditions. P2 exhibits significant variability, peaking at 84.33 MW in hour 16. P3 remains stable at a minimum output of the unit (30 MW) for many hours, with a notable increase beginning at hour 15, reaching 85.836 MW in hour 16 due to the heavy load conditions. P4 shows a rising trend, particularly after hour 7, peaking at 153.004 MW in hour 16.

P5 remains constant at 94.5 MW for most hours, with occasional drops due to lower load conditions. P6 exhibits minor fluctuations, generally around 40 MW. H5 stays consistent at approximately 29.2 MWth, except for hours 2 to 5, when it spikes to 104.8 MWth due to low power demand, indicating operation at a minimum output level that gives heat equal to 104.8 MWth. H6 remains steady at around 75 MW but experiences a drop in hour 13 due to high output from PV and WT sources. H7 presents a unique pattern, recording values of 0 during low-demand hours (2, 3, 4, and 5) or when high PV and WT output occur, such as in hour 13. In this case, the obtained results show that the overall operation cost of the studied electrical system is $247616.4.

### 5.4. Case 2

In this case, the optimal CHPEED is performed with both RESs and ESS taken into account. In the analyzed system, the optimal power and energy of the electrical energy storage system to minimize costs are 12.62 MW and 44.5 MWh. Due to its substantial power and capacity, pumped hydro storage (PHS) was selected for this investigation as it is utilized in applications demanding significant power and capacity. Information regarding it is presented in Table 5 [60].

**Table 5:** Cost and technical parameters of PHS.

Battery	Capital power cost ($/kW)	Capital energy cost ($/kWh)	Lifetime ($L_{ESS}$) (Year)	Lifecycle	Efficiency (%)
PHS	2000	50	60	40000	80

In this case, the overall operational expenses of the energy network consist of the costs linked with upgrading the energy network and the expenses related to the PHS system. The metric “Total cost of PHS per day ($)” now accounts for the distributed capital cost of the PHS, along with any future replacement costs spread out over the lifespan of the project. This adjustment captures the supplementary operating expenditures incurred by integrating the PHS into the energy network, providing a more holistic view of the total operational costs. This facilitates a fair comparison between cases with and without PHS integration. Since the lifespan of PHS is extensive, the total daily cost of PHS is inversely tied to the project duration. Extended project durations reduce daily expenses because the initial investment is spread across more operational days. In this investigation, the project is set to last 60 years with an interest rate of 0.1%. The results indicate that the total operational cost of the analyzed electrical system amounts to $245933.2 in this case. Within this total, the expenses linked to the PHS system are $27465000, with a corresponding daily cost of PHS set at $1257.1.

**Fig 15:**
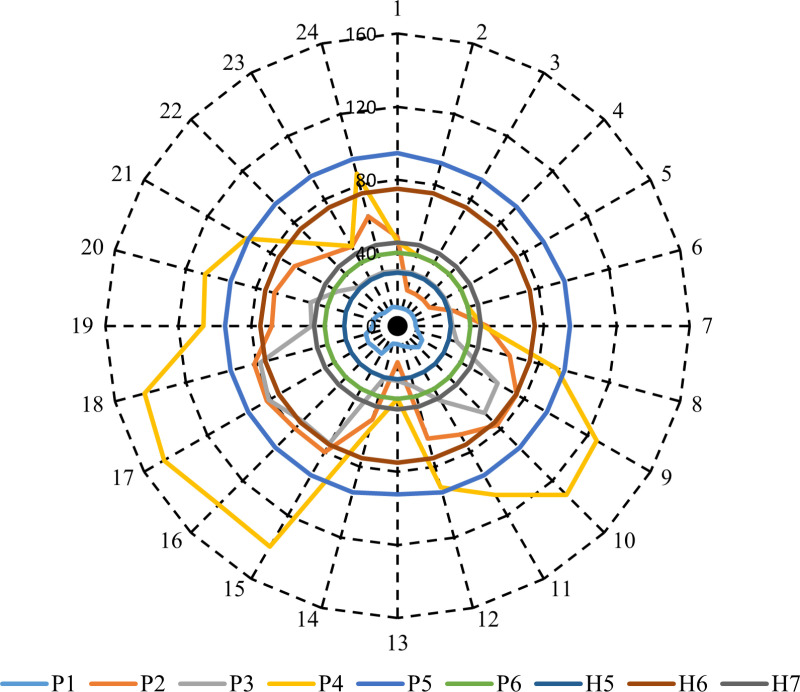
The economic dispatch for power-only units, CHP systems, and heat-only units in Case 2.

Fig 15 shows the optimal output of CHP units at each hour throughout the day to achieve minimum operation cost considering the PV units, WT system, and PHS. It can be seen from Fig 15 that the output power of the sources P1 to P6 varies at each hour depending on the electrical demand, the output power of PV and WT, the charge and discharge power of PHS, and the operational constraints of each unit, with some sources maintaining steady outputs throughout the day while others exhibit fluctuations. P1 remains relatively stable at around 10 MW for most hours, with slight increases during hours 8 to 10, suggesting minor fluctuations due to varying load conditions. P2 shows significant variability, starting at 48.7 MW in hour 1 and peaking at 82.5 MW in hour 16.

The fluctuations indicate that P2 experiences dynamic changes in response to demand or other operational factors, such as the output power of PV and WT and PHS’s charge and discharge power. P3 starts at a stable 30 MW for many hours, with notable increases beginning in hour 15, reaching a maximum of 81.251 MW in hour 16. This suggests a shift in performance during the latter part of the day due to heavy load conditions. P4 demon states a rising trend, particularly from hour 7 onward, peaking at 147.501 MW in hour 16 due to heavy load conditions. P5 remains mostly constant at 94.5 MW throughout the day, with slight variations from hour 2 to hour 5 due to the lower load and hour 13 due to the output power of RESs being high. In this case, the obtained results show that the overall operation cost of the studied electrical system is $245918.2.

**Fig 16:**
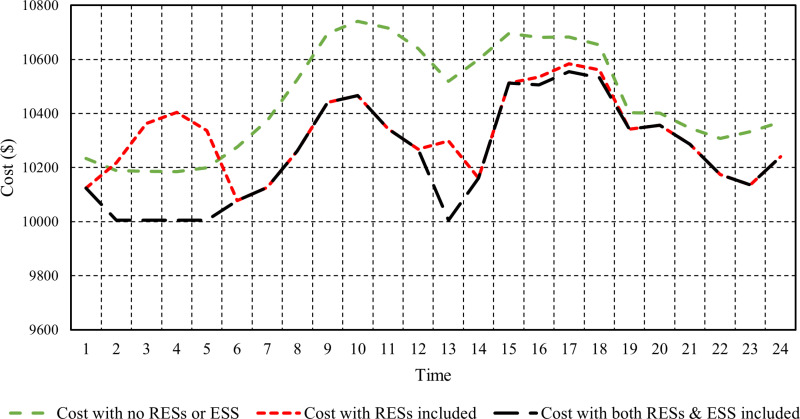
Hourly operating costs of the system in the different cases investigated.

Fig 16 illustrates the hourly operating costs of an electrical system under three different cases across 24 hours: without RESs or ESS, with RESs, and with both RESs and ESS. The case without RESs and ESS shows the highest costs, starting at approximately $10,234 and peaking at around $10,741 during hour 10 before gradually declining. In contrast, the case with RESs generally displays lower costs, ranging from about $10,124 to a peak of $10,741, indicating that using RESs contributes to reduced expenses. The case incorporating RESs and ESS consistently achieves the lowest costs, stabilizing around $10,004 for several hours, particularly early morning. This demonstrates that integrating both technologies optimizes energy usage and minimizes reliance on expensive sources, resulting in significant cost savings.

**Fig 17:**
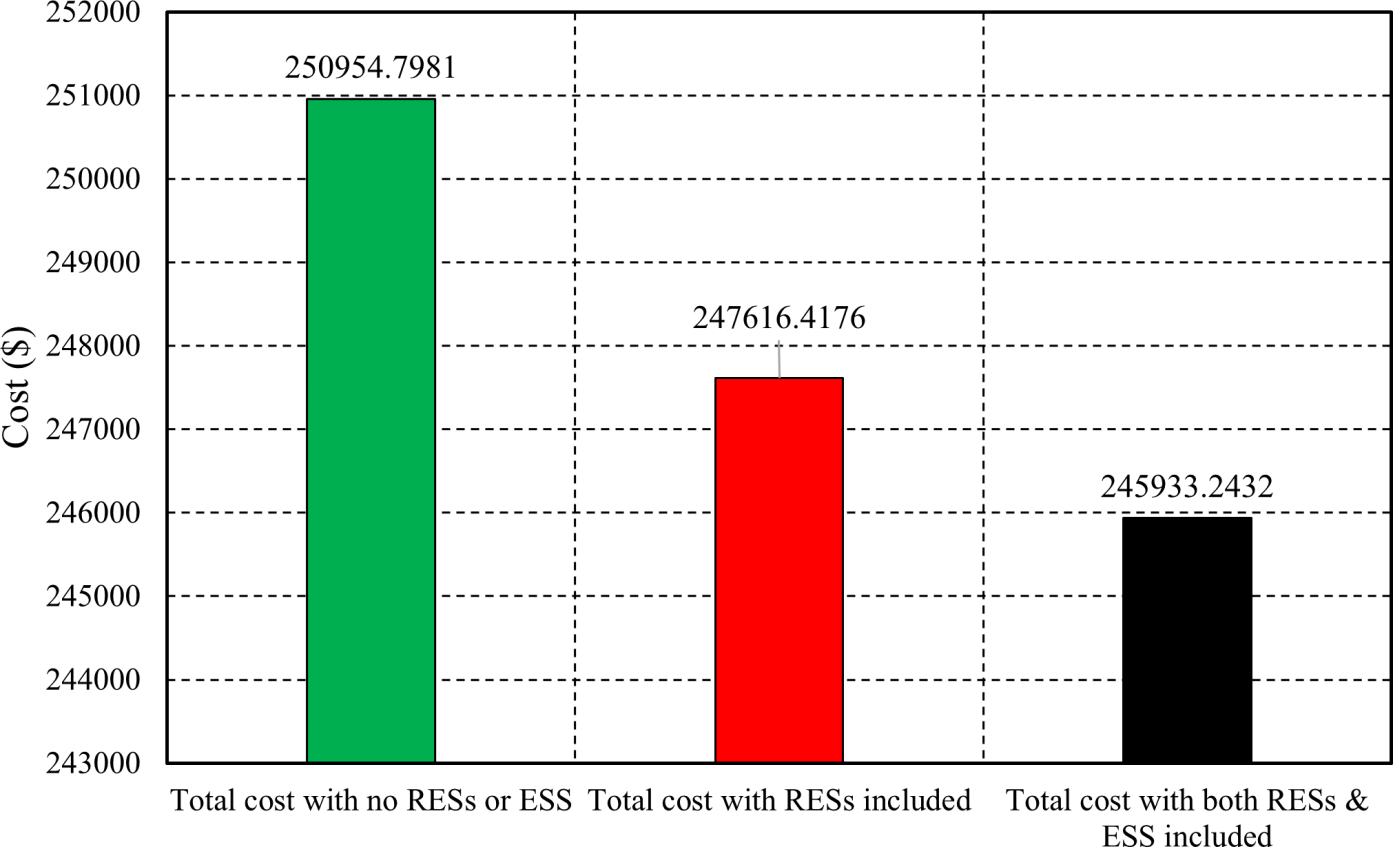
The total operation cost of the electrical system in all cases.

Fig 17 illustrates the total operating costs of an electrical system under three different cases. The case without RESs or ESS incurs the highest total cost of $250,954.80, indicating a reliance on traditional, more expensive energy sources. In contrast, the case with RESs shows a reduced total cost of $247,616.42, suggesting that integrating RESs can lead to significant savings by decreasing reliance on costly fossil fuels. The most substantial savings occur in the case that combines both RESs and ESS, costing $245,933.24. This demonstrates the effectiveness of optimizing energy usage and enhancing efficiency through RESs and ESS, allowing for better demand and supply management. Hourly cost results for the three cases for all hours are presented in the S1 file.

**Fig 18:**
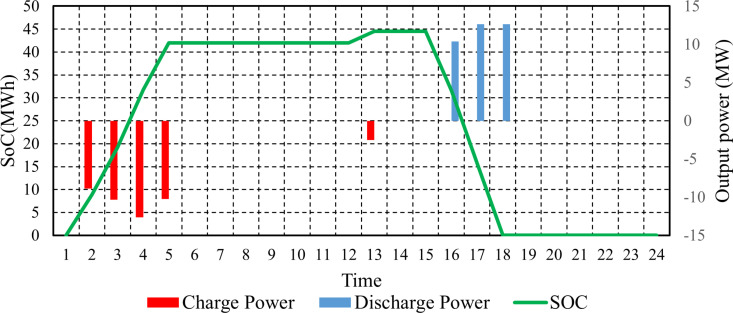
The discharge and charge power of PHS and its SoC.

Finally, Fig 18 illustrates the state of charge (SoC), charge power, and discharge power of a PHS during the day. Initially, the SoC is at zero, indicating no energy stored. As time progresses, the SoC increases, reaching a peak of approximately 44.54 MWh at several intervals, while negative values in charge power indicate that the PHS is being charged. For instance, at SoC values of 8.85 and 19.17, the PHS is charged with powers of -8.85 and -10.32 MW, respectively. However, there are periods when the battery stops charging, maintaining a constant SoC, signaling potential total capacity. Furthermore, Fig 18 reveals discharge scenarios where the SoC declines, particularly at SoCs of 31.56 and 15.78, with discharge powers of 10.38 MW and 12.63 MW, respectively. These values indicate that the PHS is supplying energy back to the system during times of higher demand.

**Fig 19:**
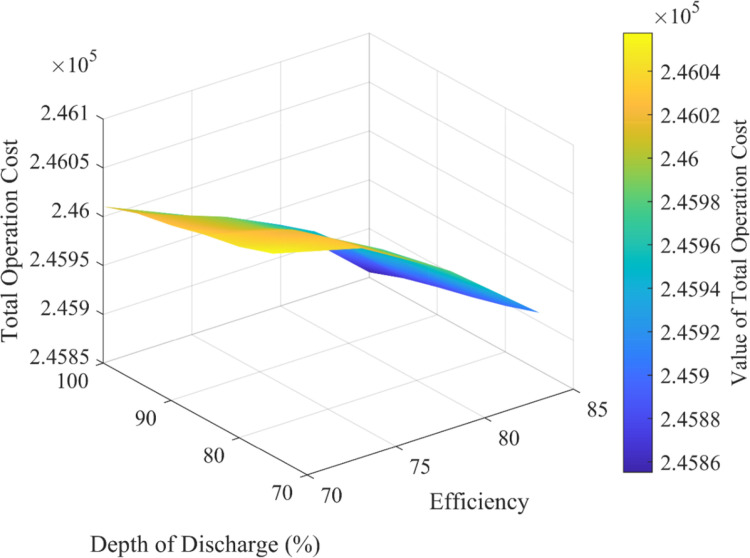
Variation of total operation cost with efficiency and DoD of PHS.

Fig 19 illustrates the relationship between total operating cost, efficiency, and depth of discharge within a system. It features three axes: the *x*-axis for efficiency, the *y*-axis for depth of discharge, and the *z*-axis for total operating cost. The figure demonstrates that total operating cost is affected by both efficiency and depth of discharge. The curvature indicates a non-linear relationship between these variables and the total operating cost. Additionally, the color bar on the right shows the total operating cost values, with a gradient reflecting variation in cost across different combinations of efficiency and depth of discharge. Darker colors typically represent higher costs, while lighter colors indicate lower costs. The analysis of Fig 19 reveals that increasing efficiency from 70% to 75% at a depth of discharge (DoD) of 100% reduces the total operating cost from $246010.5 to $245971.8. Conversely, when the efficiency is maintained at 75% and the DoD is reduced from 100% to 95%, the cost slightly increases from $245971.8 to $245989.8. These results indicate that improving efficiency generally lowers costs, while managing DoD can have varied effects, showing the importance of balancing these factors for optimal cost management.

## 6. Conclusions and future work directions

The study focuses on optimizing generation outputs to efficiently meet electricity and heat needs while minimizing costs by integrating RESs, ES, and CHP systems. A sophisticated CHPEED scheduling model is presented, which aims to minimize expenses while addressing wind and solar power variability uncertainties. Practical power system constraints and interdependencies of CHP units are navigated using fuzzy C-means for effective scenario clustering. GAMS is utilized to explore solutions comprehensively. Without RES or ESS, the cost is $250,954.80, indicating a high reliance on costly energy sources. Integrating RES reduces costs to $247,616.42, highlighting savings through decreased fossil fuel dependency. The combination of RES and ESS achieves the lowest cost of $245,933.24, showcasing improvements in efficiency and supply-demand management via optimized energy utilization. Hence, the integration of RES and ESS with CHP economic dispatch strategies presents a promising avenue for enhancing energy efficiency and sustainability. This study demonstrates that incorporating renewable resources and energy storage into the CHPEED framework can lead to further optimization, cost reduction, and decreased environmental impact.

In future works, the authors will focus on exploring the application of machine learning algorithms to enhance the predictive accuracy of renewable energy generation, investigating the impact of real-time electricity market conditions on the optimization model to improve responsiveness and adaptability, and assessing the implications of regulatory frameworks and policies on the integration of RES and ESS in CHP systems, and to further enhance sustainability, expanding the model to include more diverse energy sources and technologies, such as geothermal or biomass to conduct long-term studies to evaluate the benefits of integrating RES and ESS in various operational contexts and their impact on overall grid reliability.

## Supporting information

S1 File Hourly cost results for the three cases.
(PDF)
