# Supplementary material for: Assessing the sustainability of combined heat and power systems with renewable energy and storage systems: Economic insights under uncertainty of parameters
Source: PLoS One. 2025 Mar 18;20(3):e0319174. doi: 10.1371/journal.pone.0319174 (PMC11918332; doi:10.1371/journal.pone.0319174)
Supplement: S1 File — (PDF) [file pone.0319174.s001.pdf]

S1. Hourly cost results for the three cases.

Hour	Cost with no RES and no BSS	Cost with RES	Cost with RES and BSS
1	10233.97322	10124.29526	10124.29526
2	10188.34227	10217.98401	10004.62969
3	10185.47694	10363.21555	10004.62969
4	10184.81619	10404.2885	10004.62969
5	10199.17274	10336.599	10004.62969
6	10276.33059	10077.26781	10077.26781
7	10374.01419	10126.59758	10126.59758
8	10526.08893	10261.20488	10261.20488
9	10696.0147	10440.71882	10440.71882
10	10741.21255	10466.83793	10466.83793
11	10716.34674	10343.94695	10343.94695
12	10640.39541	10267.5415	10267.5415
13	10518.74893	10297.75564	10004.62969
14	10599.19581	10161.19141	10161.19141
15	10695.81174	10512.41007	10512.41007
16	10681.42461	10535.1301	10506.36218
17	10682.82573	10583.13495	10554.13712
18	10653.85581	10561.15703	10532.44258
19	10402.73519	10341.65409	10341.65409
20	10401.3601	10356.14399	10356.14399
21	10346.44782	10286.87378	10286.87378
22	10308.03747	10173.66341	10173.66341
23	10333.11723	10136.0351	10136.0351
24	10369.05326	10240.77032	10240.77032
Total	250954.7981	247616.4176	245933.2432
